# Facile transfer of surface plasmon electrons of Au-NPs to Zn_3_V_2_O_8_ surfaces: a case study of sunlight driven H_2_ generation from water splitting†

**DOI:** 10.1039/d3na00148b

**Published:** 2023-04-11

**Authors:** Muhammad Jalil, Khezina Rafiq, Muhammad Zeeshan Abid, Abdul Rauf, Shuxin Wang, Shahid Iqbal, Ejaz Hussain

**Affiliations:** a Institute of Chemistry, Inorganic Materials Laboratory 52S, The Islamia University of Bahawalpur Bahawalpur-63100 Pakistan ejaz.hussain@iub.edu.pk khezina.rafiq@iub.edu.pk +923026500254; b College of Materials Science and Engineering, Qingdao University of Science and Technology Qingdao-266042 P.R. China; c Department of Physics, Illinois Wesleyan University IL-61702-2900 USA

## Abstract

For future energy perspectives, an effective way to produce H_2_ from water splitting is suggested using Zn_3_V_2_O_8_ photocatalyst as a semiconductor support. Further, to enhance the catalytic efficiency and stability of the catalyst, gold metal was deposited over the Zn_3_V_2_O_8_ surface by a chemical reduction method. For comparison, the Zn_3_V_2_O_8_ and gold-fabricated catalysts (*i.e.*, Au@Zn_3_V_2_O_8_) were used for water splitting reactions. For structural and optical properties, various techniques, including XRD, UV-Vis DRS, FTIR, PL, Raman, SEM, EDX, XPS and EIS were used for the characterizations. The scanning electron microscope revealed the pebble-shaped morphology of the Zn_3_V_2_O_8_ catalyst. The FTIR and EDX results confirmed the purity and structural and elemental composition of the catalysts. Overall, 7.05 mmol g^−1^ h^−1^ H_2_ generation was observed over Au_1.0_@Zn_3_V_2_O_8_, which was ten times higher than bare Zn_3_V_2_O_8_. The results revealed that the higher H_2_ activities could be attributed to the Schottky barriers and surface plasmon electrons (SPRs). Thus the Au@Zn_3_V_2_O_8_ catalysts have potential to deliver higher hydrogen generation than Zn_3_V_2_O_8_ by water splitting.

## Abstract 2

For future energy perspectives, an effective way to produce H_2_ from water splitting is suggested using Zn_3_V_2_O_8_ photocatalyst as a semiconductor support.

## Graphical abstract

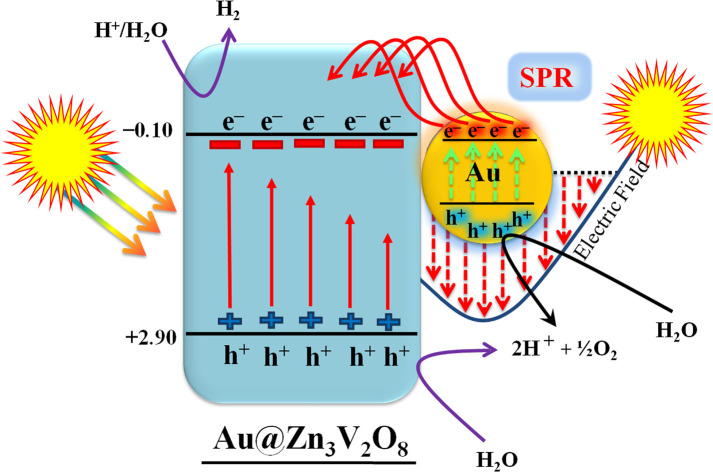

## Introduction

Industrialization and urbanization are the main factors that are leading to increase in global energy demand and associated environmental pollution. In recent decades, scientists have been seriously devoting effort towards producing alternatives to fossil fuels, which should be cheap, renewable, and carbon-free.^1^ Photocatalytic hydrogen generation from water splitting reactions meets all the current requirements. For hydrogen generation, several photocatalysts have been tried in such systems, including TiO_2_, CdS, ZnO, g-C_3_N_4,_ ZnS, WO_3,_ SrTiO_3_ and BiVO_4_.^2–9^ It has been reported that TiO_2,_ ZnO, ZnS, SrTiO_3_, and WO_3_ work in UV light due to their larger band gaps (3.2 eV, 3.3 eV, 3.6 eV, 3.2 eV, and 2.6–3.0 eV, respectively),^10^ whereas CdS, g-C_3_N_4_, and BiVO_4_ work in the presence of visible light as they have band gaps of 2.42 eV, 2.44 eV, and 2.40 eV, respectively.^11^ Although CdS is an attractive photocatalyst because it can work in visible light (due to its smaller band gap), it is subject to photocorrosion, which is the main drawback that retards its efficiency. Some recent reports have revealed that ZnCdS is a promising catalyst for hydrogen production because it has a good light corrosion resistance and responds to visible light. However, ZnCdS has limited use in photocatalytic reactions due to its low solar-energy utilization rate and fast photoelectron–hole recombination rate.^12^ Graphite-like carbon nitride (g-C_3_N_4_) photocatalysts have been extensively used for photocatalytic hydrogen production due to their narrow band gap (2.7 eV) and wide optical absorption range. However, their photocatalytic efficiency is limited because of the rapid recombination of internal charge carriers and structural defects of g-C_3_N_4_.^13^

To overcome these drawbacks of photocatalysts, scientists are working on different transition-metal vanadates due to their potential advantages, such as good stability, sufficient active states, easy band-gap engineering, presence of mixed oxidation states, non-cytotoxicity, and more available oxygen vacancies.^14^ BiVO_4_ is considered an important semiconductor due to its promising light-absorption capacity and low cost of its raw materials. However, some limitations have been reported in BiVO_4_ photocatalysts, such as poor reaction surfaces, low charge transportation, and a low conduction band level that make them unsatisfactory candidates for photocatalytic hydrogen production.^15^

Among the metal vanadates (*i.e.* BiVO_4_, CrVO_4_, FeVO_4_, Ni_3_(VO_4_)_2_, CeVO_4_), zinc vanadate (Zn_3_V_2_O_8_) has excellent optoelectronic features. To date, it has been chiefly utilized in supercapacitors, batteries, H_2_-storage devices, catalysis, photocatalysis, and magnetic devices.^16–18^ Zn_3_V_2_O_8_ photocatalysts are known to be robust against photocorrosion, inexpensive, and nontoxic, making them an attractive alternative to expensive and low-efficiency semiconductors.^19^ Due to its significant proportions of mixed oxides, Zn_3_V_2_O_8_ exhibits the potential for hydrogen-production reactions.^20^ The conduction as well as valence band of Zn_3_V_2_O_8_ are −0.10 and 2.90 eV, respectively,^21^ which are suitable for hydrogen production. Zn_3_V_2_O_8_ can be synthesized into different morphologies, including nanoparticles, nanorods, and microspheres, which can allow its use in various photocatalytic applications.^22–24^ Different methods have been adopted for the fabrication of Zn_3_V_2_O_8_, including the (i) sol–gel method, (ii) sonochemical method, (iii) hydrothermal method, (iv) chemical vapour deposition, (v) vacuum deposition method, (vi) high-energy ball-milling, and (vii) co-precipitation method.^25^

Bare Zn_3_V_2_O_8_ has a low photocatalytic efficiency for hydrogen production. Therefore, in order to enhance its activity, the use of doping or generating a hybrid Zn_3_V_2_O_8_ has been proven to be give a more active catalyst than bare Zn_3_V_2_O_8_.^26^ Recently, single-atom catalysts,^27^ and MoO_*x*_,^28^ Ru,^29^ Ag,^16,30^ Pt,^31^ and Pt/Bi alloy^32^ cocatalysts have been used to boost the photocatalytic performance of semiconductors.^27^ Metal cocatalysts represent a more practical and successful approach due to their stability, and selectivity.^33^ Gold (Au) cocatalysts have many excellent catalytic and conducting properties to boost the photocatalytic performances. Au metal cocatalysts can improve the charge separation by forming Schottky barriers and raising the Fermi levels of semiconductors.^34^ Moreover, Au expands the photoresponse range *via* an inherent surface plasmon resonance (SPR) effect.^35^

In this work, Zn_3_V_2_O_8_ photocatalysts were successfully synthesized using a co-precipitation method for hydrogen-production reactions. To enhance the hydrogen-generation activity, Au particles were incorporated over Zn_3_V_2_O_8_ surfaces; whereby the Au nanoparticles could transfer SPR electrons to the Zn_3_V_2_O_8_ support. In this work, 7.05 mmol g^−1^ h^−1^ H_2_ generation was observed over Au@Zn_3_V_2_O_8_ catalysts. Moreover, the effects of different conditions, such as pH and temperature, on the photocatalytic performances were studied. Thus, the Au@Zn_3_V_2_O_8_ photocatalysts were found to be excellent photocatalysts.

## Experimental

### Chemicals used

Ammonium metavanadate 99.95% (Sigma-Aldrich CAS Number 7803-55-6), zinc nitrate hexahydrate 98% (Sigma-Aldrich CAS Number 10196-18-6), sodium hydroxide 97% (Sigma-Aldrich CAS Number 1310-73-2), hydrochloric acid 37% (Sigma-Aldrich CAS Number 7647-01-0), tetra chloroauric acid 99% (Sigma-Aldrich CAS Number 16903-35-8), sodium borohydride 98% (Sigma-Aldrich CAS Number 16940-66-2), ethanol 95% (CAS Number 64-17-5), and distilled water.

### Synthesis

9 mmol of (2.682 g of Zn(NO_3_)_2_) solution was prepared in 30 mL distilled water. To acidify the precursor solution, a few drops of concentrated HCl were added and stirred until the contents had completely dissolved. After that, 0.702 g of NH_4_VO_3_ was added into 30 mL of 0.5 M NaOH and the resultant solution was stirred and gently heated to dissolve NH_4_VO_3_. This solution was added into the former prepared Zn (NO_3_)_2_ solution with the help of a dropper. At this stage, the pH of the reaction mixture was adjusted to 10 by adding dilute NaOH because at higher pH, more precipitates of the product are likely to be formed. After that, the mixture was stirred and heated on a hot plate at 80 °C for 3 h until white precipitates were formed. The mixture was sonicated for 10 min, and then the precipitates were allowed to settle. The precipitates were then thoroughly washed several times using an ethanol/water mixture. The products were obtained by vacuum filtration. After filtration, the precipitates were dried in an oven at 60 °C for 12 h. To enhance the crystallinity, the catalysts were then calcined at 500 °C for 5 h. Finally, the as-prepared Zn_3_V_2_O_8_ photocatalysts were saved for further use.

For the preparation of Au@Zn_3_V_2_O_8_, the as-synthesized Zn_3_V_2_O_8_ was used as a support. In a three-neck round-bottom flask, 250 mg of Zn_3_V_2_O_8_ powder was transferred and 50 mL of distilled water was added to prepare a homogeneous slurry. Then, this slurry was sonicated for 15 min and stirred for about 1 h. About 337 mg of HAuCl_4_ was added to the above slurry. Then this precursor slurry was purged with pure argon gas to remove the oxygen contents. NaBH_4_ solution was added for the *in situ* reduction of Au metal ions, followed by continuous stirring at room temperature. The metal-supported mixture was sonicated again for 20 min and transferred into a 100 mL Teflon line stainless steel autoclave reactor for hydrothermal treatment. The temperature of the hydrothermal reaction was fixed at 180 °C for about 10 h. After the completion of the hydrothermal reaction, the product was filtered and thoroughly washed with distilled water and ethanol. At the final stage, the catalyst precipitates were recovered *via* centrifugation. The catalysts were dried in an oven at 90 °C for 3 h. The obtained fine powder catalysts were ground using a mortar and pestle. The powder was then calcined at 400 °C to improve the crystallinity and purity of the product. The catalysts were finally collected for characterization and for later use in the hydrogen-evolution experiments.

### Characterizations of the catalysts

To obtain information on the structural morphology of the bare Zn_3_V_2_O_8_ and Au@Zn_3_V_2_O_8_, X-ray diffraction (XRD) patterns were collected on a Bruker D2 Phaser instrument (LUMS, Lahore Scientific Instrument Laboratory). Fourier-transform infrared (FTIR) characterizations were performed on a Bruker Alpha Platinum ATR system (Spectral Range: 7500–375 cm^−1^). RAMAN spectroscopy was performed on a StellerNet Raman high-resolution spectrometer. Scanning electron microscopy (SEM) with energy dispersive X-ray (EDX) analysis was performed on an FEI Nova 450 Nano SEM system. Atomic force microscopy (AFM) was performed using a PARK NX10 instrument. Ultraviolet-visible-diffuse reflectance spectroscopy (UV-vis-DRS) was performed on a UV-Vis spectrophotometer SP-IUV7 (wavelength range: 190–1100 nm, Wavelength accuracy: ±0.5 nm). Photoluminescence (PL) analysis was performed on an MFLI Lock-in Amplifier (bandwidth = 500 kHz). X-Ray photoelectron spectroscopy (XPS) analysis was performed on a Kratos Axis ultra-DLD spectrometer equipped with an Al X-ray radiation source. A Solatron impedance analyzer was used for the EIS analysis (amplitude = 10–100 mV, frequency range = 0.1 Hz–1 MHz). Photocurrent tests were carried out in a conventional three-electrode system using a potentiostat (CH Instruments, CHI 660) and visible-light irradiation (*λ* > 420 nm). Gas chromatography (GC-TCD) was used to determine the hydrogen-evolution activities of the as-prepared Zn_3_V_2_O_8_ and Au@Zn_3_V_2_O_8_ photocatalysts.

### Hydrogen-evolution experiments

The photocatalytic reactions for hydrogen production were run in a Pyrex reactor (140 mL) in the presence of sunlight. Here, 5 mg of photocatalysts were loaded in the reactor containing a 25 mL mixture of 5% ethanol and 95% water. Prior to the reaction, the reactor was continuously bubbled with highly pure nitrogen gas at a flow rate of 12 mL min^−1^ for 45 min in order to remove the oxygen content from the previous reaction mixtures in the reactor. Sunlight was used as the irradiation source for the activation of the photocatalysts. The catalysts were irritated with light having a photon flux of *ca.* 6.5 mW cm^−2^, which approximates to the photon flux of solar light. The gas sample was collected by a 0.5 mL syringe (UK standard) from the gas head space of the photoreactor and transferred to the gas chromatograph (GC-TCD) containing a molecular sieve capillary column (length = 25 mm, average thickness = 0.5 μm) and a TCD detector. An internal calibration curve was used to quantify the H_2_ produced by the photocatalytic reaction. Photocatalytic tests for the each sample were repeated at least three times. The hydrogen-evolution rates were measured in mmol g^−1^ and mmol g^−1^ h^−1^ to allow comparisons between different photocatalysts under similar conditions.^36^ The quantum yield of the photocatalysts was measured by the ratio of number of H_2_ molecules produced to the number of photons captured by the photocatalysts, as per the following formula.
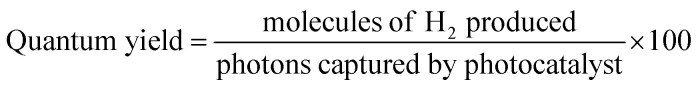


## Results and discussion

**Fig. 1:**
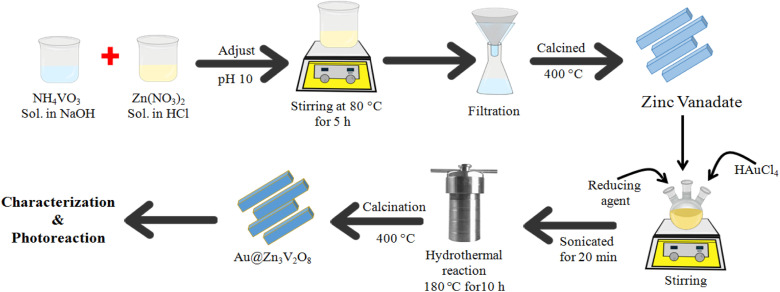
Illustration of the synthesis of the Zn_3_V_2_O_8_ and Au@Zn_3_V_2_O_8_ catalysts.

Fig. 1 illustrates the synthesis scheme employed to prepare the Zn_3_V_2_O_8_ and Au@Zn_3_V_2_O_8_ photocatalysts (details are given in the Experimental section). To enhance the crystallinity and purity, all the photocatalysts were calcined at 400 °C for 4 h.

### XRD

**Fig. 2:**
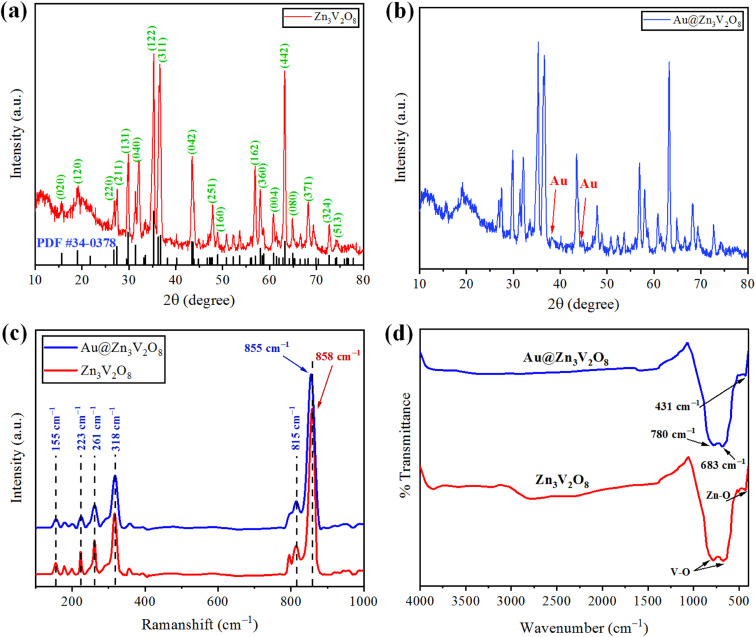
(a and b) XRD patterns of Zn_3_V_2_O_8_ and Au@Zn_3_V_2_O_8_ catalysts. (c and d) Raman and FTIR results of pure Zn_3_V_2_O_8_ and Au@Zn_3_V_2_O_8_, respectively.

The crystallinity and phase purity of pure Zn_3_V_2_O_8_ and Au@Zn_3_V_2_O_8_ were examined by X-ray diffraction (Bruker D2 Phaser). The XRD patterns were collected in the 2 theta range between 10–80°. The XRD patterns of the as-synthesized Zn_3_V_2_O_8_ are illustrated in Fig. 2(a), and were in agreement with JCPDS card number 34-0378. The peaks were observed at 15.365°, 18.741°, 26.450°, 27.080°, 29.454°, 31.004°, 34.910°, 35.980°, 36.464°, 43.114°, 43.301°, 48.606°, 57.703°, 58.440°, 60.562°, 62.973°, and 64.606°, corresponding to the *HKL* values (020), (120), (220), (211), (131), (040), (122), (320), (311), (042), (151), (160), (162), (360), (004), (442), and (080), respectively. The crystallite size of the Zn_3_V_2_O_8_ was determined by using Scherrer's equation as 14.95 nm. Calculations for the crystallite size are provided in Table S1.† The crystal structure of Zn_3_V_2_O_8_ was orthorhombic with 4.83 g cm^−3^ density. All the peaks confirmed the XRD pattern of pure Zn_3_V_2_O_8_ material. The intensity could be increased or decreased by increasing or decreasing the concentration of the sample to be examined; whereby a high peak intensity reflects the efficiency of the instrument. In the XRD patterns, some distinct peaks assured the presence of gold at the Zn_3_V_2_O_8_ surfaces Fig. 2(b). The XRD pattern of Au@Zn_3_V_2_O_8_ presented the peak of Au at 38.18°, corresponding to the *hkl* value (111) evidenced by JCPDS card number 04-0784. The average crystallite sizes of the Au@Zn_3_V_2_O_8_ catalysts were observed as 18.80 nm (using Scherrer's formula). The XRD parameters are presented in Table S2.†

### Raman analysis

The vibration modes of the molecules were determined by the Raman spectroscopy technique. The rotational and other low-frequency modes of the molecules were also computed. Fig. 2(c) presents the Raman spectrum of the as-synthesized Zn_3_V_2_O_8_ catalysts in the 100–1000 cm^−1^ scan range. In the spectrum, significant peaks could be observed at 155, 223, 261, 318, 373, 815, and 907 cm^−1^. Bending vibrations (V–O) and lattice modes were observed in the 100–315 cm^−1^ range. The peaks at 318 cm^−1^ (lower wave number) represented the second-order Raman scattering mode, while the peak at 373 cm^−1^ represented the first-order Raman scattering modes and arose because of some defects (*i.e.* oxygen vacancies). Similarly, the few peaks observed at 155 cm^−1^ were attributed to the stretching modes of vanadium oxides lattices. A broad peak observed at 261 cm^−1^ was due to the bending mode of vanadium oxide. In the 740–1000 cm^−1^ range, distinct modes could be observed due to the different oxidation states of vanadium.^37^ In the case of Au@Zn_3_V_2_O_8_ catalysts, a shift in the most intense peak was observed (towards lower wave number) from 858 to 855 cm^−1^. The shift in the case of the gold-deposited catalysts was due to a phonon confinement effect and due to the defects in the Zn_3_V_2_O_8_ lattices^38^ (see the results in Fig. 2(c)).

### FTIR study

Fourier-transform infrared (FTIR) spectroscopy was performed and substantiated the linkage between the atoms of the Zn_3_V_2_O_8_ catalysts. Fig. 2(d) shows the FTIR spectrum of the synthesized Zn_3_V_2_O_8_ catalysts in the scanned range from 400–4000 cm^−1^. Two peaks appeared at 431 and 435 cm^−1^, which were due to stretching vibrations of Zn–O.^39^ Similarly, two peaks of V–O vibrations appeared at 683 and 780 cm^−1^ corresponding to the tetrahedron of the VO_4_ vibrational modes. The appearance of vibrations at 3000 cm^−1^ was attributed to O–H stretching from the moisture contents in the catalysts. In the FTIR results, no distinct peak of Au metals appeared because of low percentage at the Zn_3_V_2_O_8_ surfaces.

### SEM with EDX

**Fig. 3:**
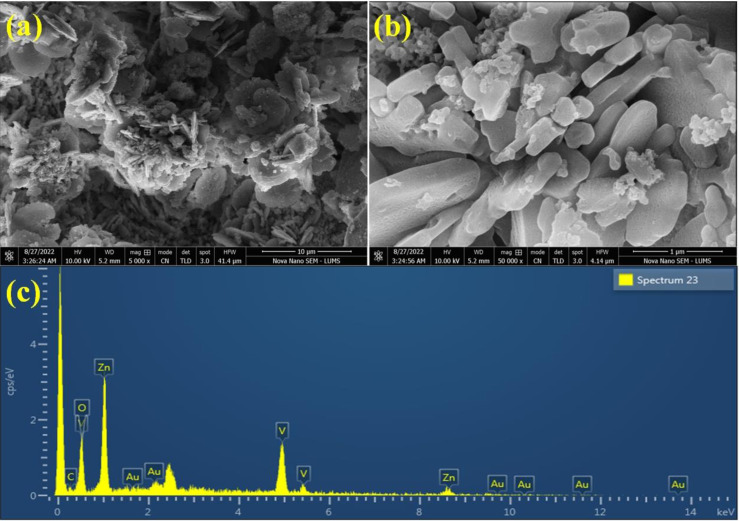
SEM images of the catalyst at (a) 10 μm and (b) 1 μm. (c) EDX results of the catalyst.

The SEM images of Au@Zn_3_V_2_O_8_ demonstrated a pebble-like nanoplates morphology, as shown in Fig. 3(a) and (b). The formation of nanoplates was achieved by several processes; first zinc combined with vanadium to form zinc vanadate nuclei. After that, the growth of nanoplates started, followed by the exfoliation process, and finally the self-aggregation of zinc vanadate occurred.^40^ In the SEM results, it could be observed that the morphology of the as-synthesized Zn_3_V_2_O_8_ depended on the temperature at which sample was calcined or at which the hydrothermal reaction took place. It could be observed that during the catalysts preparations, the concentration of the precursor solutions and pH of the reaction mixture were important factors. For example, at high temperature, the zinc vanadate nanoplates split into thin sheets; these sheets underwent exfoliation, resulting in a higher porosity in the lattices. The elemental composition of the Au@Zn_3_V_2_O_8_ catalysts was illustrated by EDX. The EDX results demonstrated that the Au particles were homogeneously dispersed on the zinc vanadate surfaces. Moreover, the EDX results confirmed the existence of Zn, V, and O, clearly verifying the purity of the catalysts, as shown in Fig. 3(c).

### XPS

**Fig. 4:**
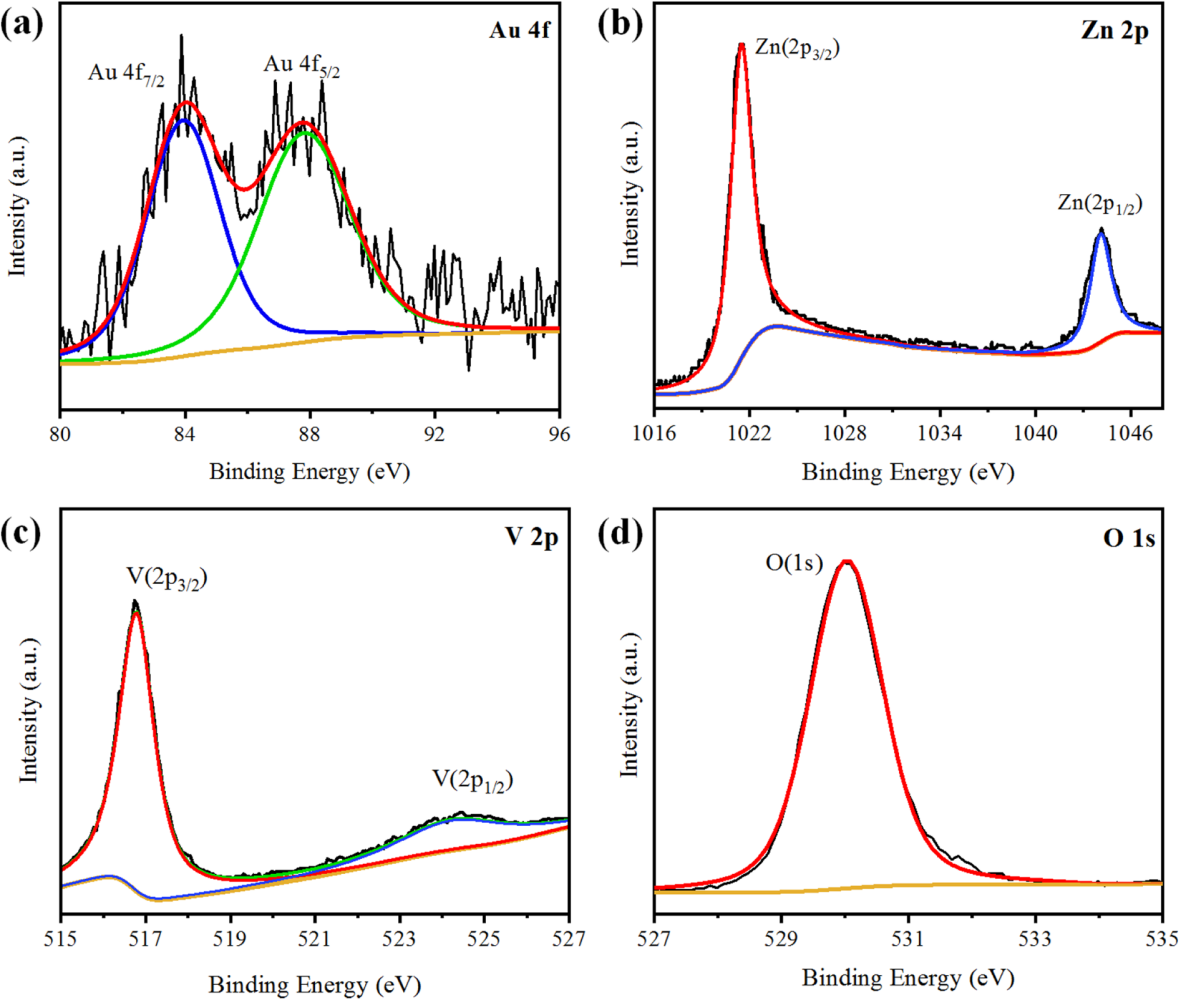
XPS results of Au@Zn_3_V_2_O_8_ showing: (a) Au 4f, (b) Zn 2p, (c) V 2p, and (d) O 1s plots.

XPS analysis was performed to analyse the chemical composition and oxidation states of the elements present in the Au@Zn_3_V_2_O_8_ photocatalysts. The XPS results of Au@Zn_3_V_2_O_8_ are shown in Fig. 4(a)–(d). The XPS results for Au indicated the presence of two peaks at 84.08 and 87.78 eV, attributed to Au 4f_7/2_ and 4f_5/2_, respectively, as shown in Fig. 4(a). The Zn 2p_3/2_ and Zn 2p_1/2_ peaks were observed at 1021.45 and 1044.25 eV, respectively, as depicted in Fig. 4(b). According to the high-resolution spectrum of vanadium, two peaks of 2p were observed at 516.79 and 524.17 eV, which were attributed to V 2p_3/2_ and V 2p_1/2_, respectively; see Fig. 4(c). An oxygen O 1s peak appeared at 529.98 eV (Fig. 4(d)), which was assigned to the oxygen lattice in the Au@Zn_3_V_2_O_8_ photocatalyst.^16^

### AFM

**Fig. 5:**
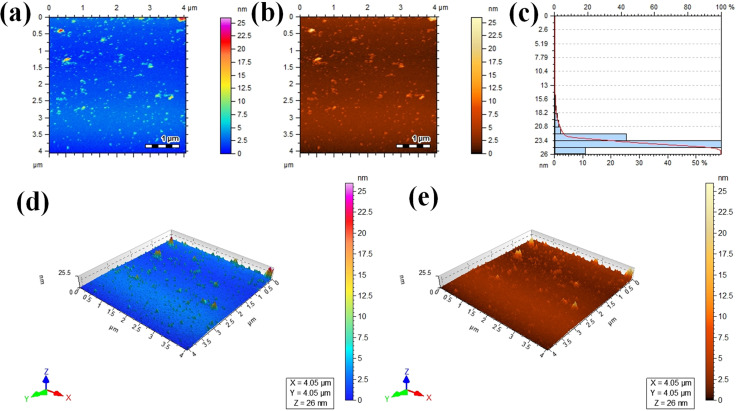
AFM results: (a and b) 2D tapping modes, (c) size distribution, and (d and e) 3D images of the Au@Zn_3_V_2_O_8_ photocatalysts.

Atomic force microscopy (AFM) was used to assess the informations about thickness and height of the Au@Zn_3_V_2_O_8_ photocatalysts. Two dimensional (2D) AFM images of the photocatalysts are shown in Fig. 5(a) and (b), and the height of the photocatalysts was 25.5 nm as presented in Fig. 5(c). The three-dimensional (3D) view of the photocatalysts height is clearly demonstrated in Fig. 5(d) and (e). The scan areas for the measurement of the thickness and height of photocatalysts were 4.05 × 4.05 μm and 0–26 nm, respectively. The AFM images showed that the photocatalysts particles had rough surfaces and a homogenous distribution of gold particles. The height and morphology of the photocatalysts as exposed by the atomic force spectroscopy (AFM) and SEM analyses were consistent with the previously reported work.^41^

### UV-Vis-DRS

**Fig. 6:**
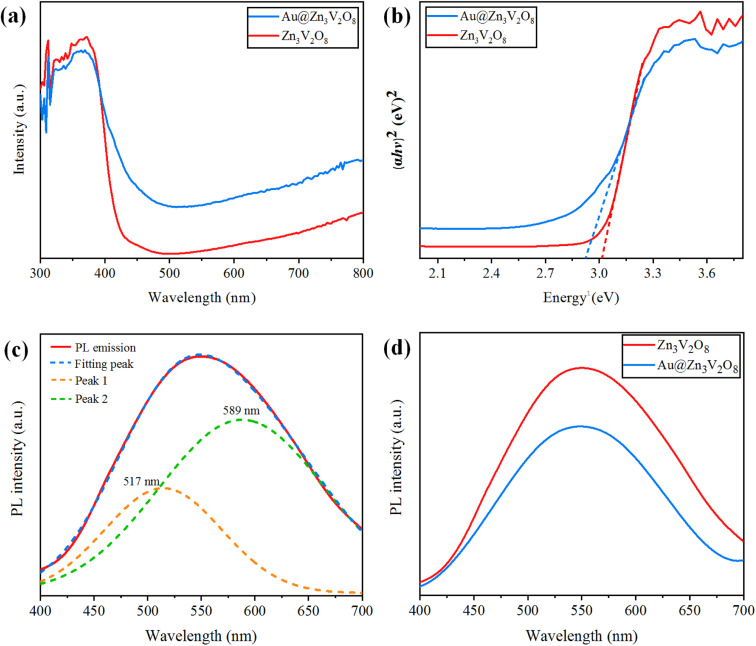
(a) UV-Vis-DRS, (b) energy curves (eV), (c) PL emission spectra and fitting of the peaks, and (d) PL emission spectra for the Z_3_V_2_O_8_ and Au@ Z_3_V_2_O_8_ catalysts.

Ultraviolet-visible-diffuse reflectance spectroscopy (UV-Vis-DRS) gives information on the band gap, charge separation, and optical properties of photocatalytic materials. The UV-Vis-DRS results for Zn_3_V_2_O_8_ and Au@Zn_3_V_2_O_8_ are exhibited in Fig. 6(a). The calculated band gaps of pure Zn_3_V_2_O_8_ and Au@Zn_3_V_2_O_8_ were 3.0 and 2.9 eV respectively, see Fig. 6(b). The absorbance edge of pure Zn_3_V_2_O_8_ was observed at 413 nm, whereas the presence of Au on the surfaces of Zn_3_V_2_O_8_ extended the absorbance to the visible region of the solar spectrum (*i.e.* 427 nm). Higher activities were attributed due to extended absorption and greater electron transfer from the oxygen 2p orbital to the vanadium vacant 3d orbital in the VO_4_ tetrahedron. Furthermore, Au provided SPR electrons to facilitate the excited electrons to the redox centres of the VO_4_ tetrahedron.^42^

### Photoluminescence (PL)

Photoluminescence (PL) spectra can be used to explain the charge separation and recombination rate of photocatalytic semiconductors used for photocatalytic reactions.^43^ If the intensity of PL emission is high, this represents that there will be a higher electron recombination and low efficiency of photocatalysts during photoreactions; whereas if the intensity of the PL spectra is low, then the recombination rate will be low and as a result, the photocatalytic efficiency will be high. When the photocatalytic efficiency is high, then there will be more electrons in the conduction band and the hydrogen-production rate will increase significantly. Here, it was observed that the existence of Au metals at the zinc vanadate surfaces generated more electrons, and as a result more hydrogen was produced. Due to the inherent SPR in Au metal particles, the excitation of electrons from the gold core will be easier, which can then be energetically promoted to the conduction band of zinc vanadate. The PL spectra of the synthesized Zn_3_V_2_O_8_ and Au@Zn_3_V_2_O_8_ are shown in Fig. 6(c) and (d). In Fig. 6(c), the emission spectra showed a wide band from 450 to 700 nm; this emission band further implied two peaks that were centred at 589 and 517 nm. These peaks were Gaussian emission peaks,^44^ that is why Zn_3_V_2_O_8_ exhibited a yellow-light emission in the 450–700 nm range. Fig. 6(d) clearly demonstrates the emission spectra of the Zn_3_V_2_O_8_ and Au@Zn_3_V_2_O_8_ catalysts. The emission spectra of the Zn_3_V_2_O_8_ catalysts were due to the charge recombination/back reactions. The PL results agreed with the UV-Vis-DRS results and the photocatalytic activities (see the Activity section).

### Photocurrent response and electrochemical impedance spectroscopy (EIS)

**Fig. 7:**
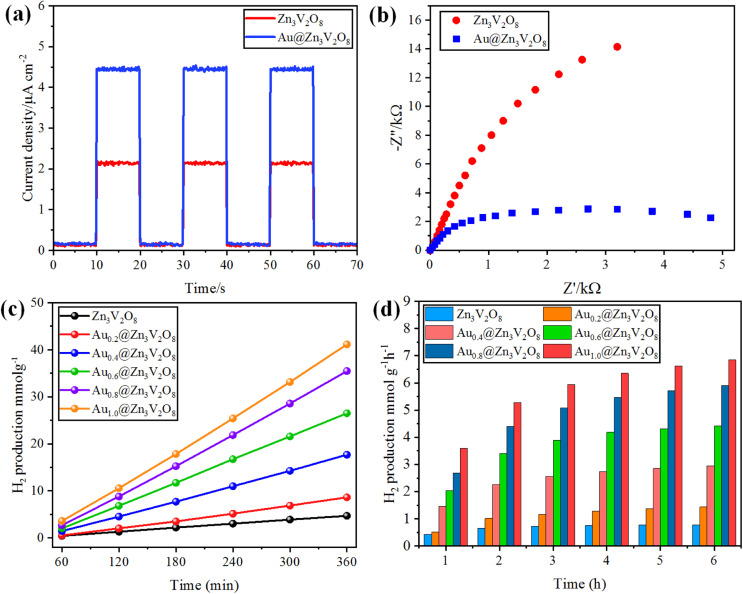
(a) Photocurrent response, (b) EIS results of Zn_3_V_2_O_8_ and Au@Zn_3_V_2_O_8_. (c and d) comparison of the photocatalytic activities of the photocatalysts in mmol g^−1^ and mmol g^−1^ h^−1^, respectively.

The photocurrent response and EIS analyses were used to analyse the charge transfer and transport processes. Pure Zn_3_V_2_O_8_ and Au@Zn_3_V_2_O_8_ were analyzed for their photocurrent responses with on/off cycles under visible-light irradiation. The Au@Zn_3_V_2_O_8_ photocatalysts exhibited a higher photocurrent density compared to pure Zn_3_V_2_O_8_ and they were found to be stable, as demonstrated in Fig. 7(a). The results demonstrated that Au@Zn_3_V_2_O_8_ had a high separation efficiency for electrons and holes. The EIS analysis results demonstrated a much smaller diameter of the arc radius than for the pure Zn_3_V_2_O_8_, Fig. 7(b). The results reveal that the Au@Zn_3_V_2_O_8_ photocatalyst had a low interfacial charge resistance and fast interfacial charge-transfer efficiency. Overall, the use of PL, photocurrent response, and EIS analyses provided a basis for understanding the performance of the Au@Zn_3_V_2_O_8_ photocatalysts.^45^

### Hydrogen-evolution activities

To determine the hydrogen evolution, the as-synthesized photocatalysts were used under visible-light irradiation with adding 5% ethanol as a sacrificial agent. The total time for the photocatalytic reaction was fixed and optimized at 6 h for each photocatalyst tested. Hydrogen evolution occurred only by photocatalytic reaction, and no hydrogen was produced in the absence of irradiation and catalysts, and even a large amount of Zn_3_V_2_O_8_ could produce only a meager amount of hydrogen due to the fast recombination of holes and electrons. Au metal was loaded on the Zn_3_V_2_O_8_ support with different wt% of metal to prevent the recombination of electrons and enhance the hydrogen production.^20^ By increasing the Au wt% from 0.2 to 1.0 wt%, the effect of the metal loading was observed on the hydrogen-evolution rate, which increased with the increase in metal loading. Specifically, the hydrogen-evolution activities of Zn_3_V_2_O_8_, Au_0.2_@Zn_3_V_2_O_8_, Au_0.4_@Zn_3_V_2_O_8_, Au_0.6_@Zn_3_V_2_O_8_, Au_0.8_@Zn_3_V_2_O_8_ and Au_1.0_@Zn_3_V_2_O_8_ were 4.69, 8.61, 17.52, 27.09, 36.36, and 42.31 mmol g^−1^, respectively, as shown in Table 1 and Fig. 7(c) and (d). Zn_3_V_2_O_8_ loaded with 1.0 wt% of Au exhibited 42.31 mmol g^−1^ hydrogen evolution, which was the highest activity among all the catalysts. The maximum wt% of Au metal loaded on Zn_3_V_2_O_8_ was 1.0%, which produced 7.05 mmol g^−1^ h^−1^ of hydrogen by water splitting reactions. Au_1.0_@Zn_3_V_2_O_8_ was observed to be the most active photocatalyst in this study. It has been observed that Au metal plays a vital role in enhancing the hydrogen evolution. Moreover, due to the transfer of surface plasmon resonance (SPR) electrons to the redox sites of the catalysts, higher hydrogen generation can be achieved. Herein, 1.0 wt% Au loading was optimized for the Pyrex 140 mL reactor system. It was also found that an increased Au loading on Zn_3_V_2_O_8_ from 1.0 to 1.4 wt% did not affect the hydrogen-evolution rate much. This was because increasing the Au weight percentage caused the co-catalyst particles to aggregate and form large clusters, which inhibited the transfer of charge carriers to the active sites of the photocatalyst.^46^ The hydrogen-production activity also increased due to the stability gained by the gold deposited over the Zn_3_V_2_O_8_ surface. To verify the SPR effect of the Au metal contents over Zn_3_V_2_O_8_, band pass filters of 420, 500, 600, and 700 nm (centred at 20 nm) were used. The H_2_-production rates (mmol g^−1^ h^−1^) from C_2_H_5_OH/H_2_O mixtures (1 : 19) are illustrated in Fig. S1.† At 700 and 600 nm, hydrogen was observed in trace amounts because of the minute quantity of charge carriers produced. Moreover, at 500 nm, the H_2_-production rate accelerated due to the SPR electrons of the Au metal contents.^47^ However, no H_2_ was detected for the bare Zn_3_V_2_O_8_ photocatalyst under the same reaction conditions. In comparison to bare Zn_3_V_2_O_8_, Au@Zn_3_V_2_O_8_ exhibited a H_2_-production ability under visible light, indicating that the Au content over Zn_3_V_2_O_8_ led to H_2_ production due to the SPR electrons. Several photocatalysts have been reported for the production of hydrogen by photocatalytic water splitting. The hydrogen-generation results for some reported catalysts are listed in the ESI in Table S3.†

**Table 1:** Comparison of the hydrogen-evolution activities of various as-prepared catalysts^a^

S. no.	Photocatalysts	Au at the support (wt%)	Reaction time (6 h)	H_2_ evolution	
				mmol g^−1^	mmol g^−1^ h^−1^
1	Zn_3_V_2_O_8_	0.0	10:00 am to 16:00 pm	4.69	0.78
2	Au_0.2_@Zn_3_V_2_O_8_	0.2	10:00 am to 16:00 pm	8.61	1.43
3	Au_0.4_@Zn_3_V_2_O_8_	0.4	10:00 am to 16:00 pm	17.52	2.95
4	Au_0.6_@Zn_3_V_2_O_8_	0.6	10:00 am to 16:00 pm	27.09	4.51
5	Au_0.8_@Zn_3_V_2_O_8_	0.8	10:00 am to 16:00 pm	36.36	6.06
6	Au_1.0_@Zn_3_V_2_O_8_	1.0	10:00 am to 16:00 pm	42.31	7.05

a Amount of catalysts = 5 mg, sacrificial reagent = 5% ethanol, photon flux = sunlight/*ca.* 6.5 mW cm^−2^.

### Mechanism

**Fig. 8:**
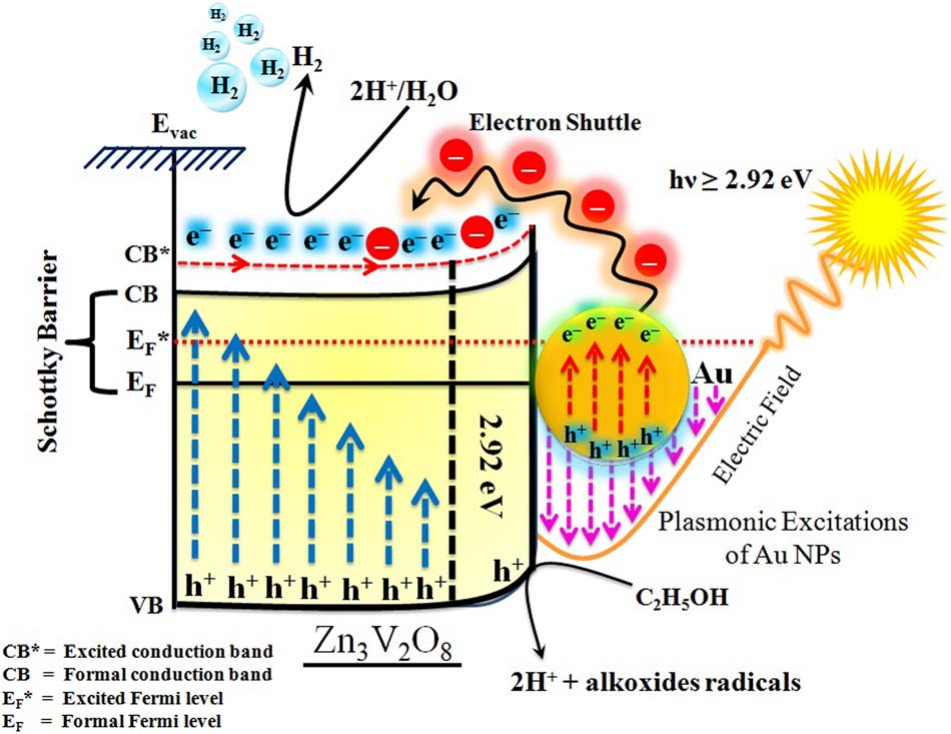
Illustration of SPR electron transfer for photocatalytic H_2_ evolution.

The Gibbs free energy required for water splitting is 237.2 kJ mol^−1^ (2.46 eV) and so photocatalysts with proper band structures can only progress this reaction.^48^ The overall water splitting reaction involves two half reactions: first reduction and then oxidation. For efficient water splitting, the conduction band potential of photocatalysts should be more negative than the hydrogen reduction potential to carry out the reduction half reaction. Similarly, for a successful oxidation half reaction, the valence band potential of the catalysts should be more positive than the oxidation potential of water, which is 1.23 eV.^36^ It is well-known that the particle size, and chemical and physical properties are changed by changing the energy gap (*E*_g_) of photocatalysts. During water splitting along with hydrogen generation, oxygen is not always generated due to the existence of sacrificial reagents. The sacrificial reagents consume the holes (h^+^) present at the surfaces of the photocatalysts. Due to the irradiation of sunlight, electrons are excited and moved to the conduction band (CB) of photocatalysts. Due to its higher work function, Au metal has the ability to quench these electrons and utilize them for the reduction of aqueous H^+^.^49^ The hydrogen atoms start to generate H_2_ molecules when they combine with each other. Moreover, gold metal centres cause the developments of a junction between Zn_3_V_2_O_8_ and the cocatalysts, which prevents the recombination of electrons with holes at the reaction surfaces. The other advantage of gold metal is that it causes a surface plasmon impact during the photoreaction. Due to the inherent surface plasmon characteristics of gold, the rate of the water reduction reaction is significantly enhanced.^50^ Under sunlight irradiation, electrons from the Au metal get induced by surface plasmon resonance (SPR), as shown in Fig. 8, and provide a Schottky junction at the point of contact between the metal and semiconductor.^51,52^ During the photoreaction, the Fermi level (*E*_F_) of Zn_3_V_2_O_8_ is raised up due to the movement of the Schottky barrier electrons at the surfaces. It has been reported that, due to reinforcement of the Fermi energies, electron transfer to the conduction band level becomes more feasible and from where they get utilized for water reduction.^53^ When excited electrons arrive at the conduction band, then the Fermi energy level (*E*_F_) is reinforced and is then known as the excited energy Fermi level 
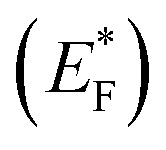
.^54,55^ Due to the new Fermi energy levels, the energy of conduction band also increases and new excited conduction bands (CB*) are formed. These excited conduction bands lie below the vacuum level (*E*_vac_) of the semiconductors, resulting in higher activities.

### Recyclability

It is important for catalysts that they can be reused and recycled after photocatalytic reactions, which is directly associated with their stabilities.^56^ Au@Zn_3_V_2_O_8_ was found to be a stable catalyst that can be reused for the production of hydrogen from the photocatalytic water splitting reaction. Once the photocatalytic splitting of water was completed, the Au@Zn_3_V_2_O_8_ photocatalysts did not degrade and they were collected for another reaction. A diagram presenting the recyclability tests carried out for Au@Zn_3_V_2_O_8_ is included in the ESI (see Table S4 and Fig. S2†). After each photocatalytic reaction, the photocatalysts were recovered by centrifugation and washed with distilled water and dried in an oven at 90 °C for 3 h before reuse. Only a minor decrease in the hydrogen-generation yield was found, attributed due to the loss of photocatalysts during drying and washing.

### Factors affecting the activity of the photocatalysts for hydrogen production

Several factors influence the activity of photocatalysts for hydrogen generation, namely the band gap, intensity of light, surface area, pH, temperature, and sacrificial agents. In this study, the following describes the factors that were evaluated for assessing the photocatalyst performance.

### Band gap

The difference in energy between the energies of the valence band and conduction band of a semiconductor is regarded as the band gap. Hydrogen gas is produced by the reduction of H^+^ on the active sites of the catalysts. For water splitting, the valence band potential of photocatalysts should be more positive than the oxidation potential of O_2_/H_2_O while the conduction band potential should be more negative than the reduction potential of H_2_O/H_2_. Catalysts with a band gap of 3.2 eV or more can split water only in the presence of UV irradiation, but in visible light they are not effective for hydrogen evolution. To enhance the activity in visible light, metal loading/doping is mandatory to reduce the band gap, which can make a catalyst more effective at producing hydrogen under visible irradiation. To reduce the band gap of Zn_3_V_2_O_8_, Au metals were loaded on it to change the band gap to 2.9 eV, which is a more suitable band gap for the evolution of hydrogen in the presence of visible irradiation. Thus, Au-loaded Zn_3_V_2_O_8_ produced more hydrogen compared to the bare Zn_3_V_2_O_8_ due to the difference in band gap energy. For the band gaps, see Fig. 6(b).

### Intensity of light

**Fig. 9:**
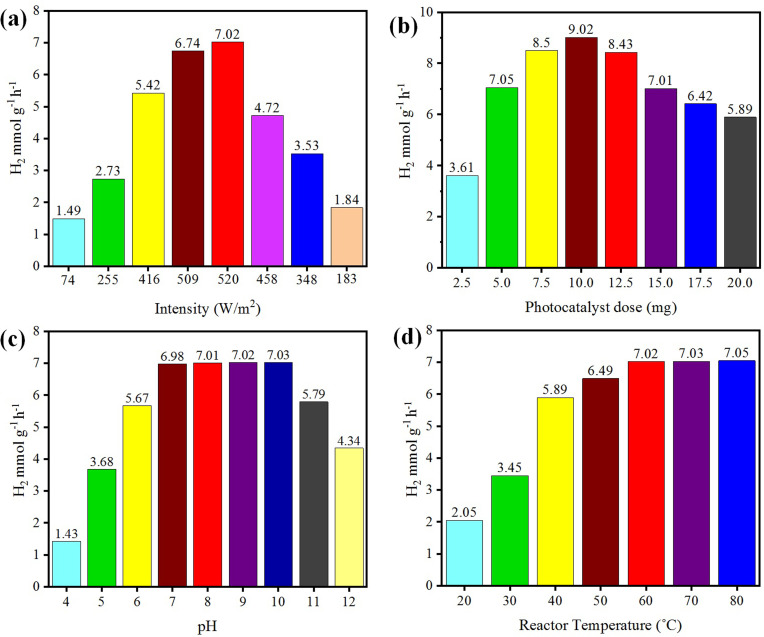
Effects of (a) intensity, (b) concentration, (c) pH, and (d) temperature on photocatalytic H_2_ generation.

The photoreaction for the generation of hydrogen was carried out in the presence of sunlight. The photocatalysts activity increased with the increase in light intensity. The intensity of sunlight above the surface of the earth is approximately 1380 W m^−2^. In the morning (*i.e.* 7:00 to 10:00 am), the rate of hydrogen evolution was low due to the low activation of photocatalysts with the low intensity of light penetration to the surfaces of catalysts; whereas after 10:00 am, the hydrogen-evolution rate increased due to the higher intensity of light directly hitting the photocatalysts. Then, after 4:00 pm the activity of the catalysts decreased due to the low light intensity as the sun set. It was observed that, at evening time, the photocatalysts again became less active for hydrogen evolution. Several runs were performed to examine the effect of the intensity of sunlight irradiation to monitor the variation of photocatalytic activity. It was found that, as the intensity of light increased, the hydrogen-evolution rate also increased, as shown in Fig. 9(a). A maximum 7.02 mmol g^−1^ hydrogen evolution was recorded under sunlight irradiation with an intensity of 520 W m^−2^.

### Concentration

The concentration of the photocatalysts was found to be another factor that can affect the activity of the photocatalysts. As the concentration of photocatalyst increased, its exposure to the sunlight also increased and more electrons were produced, resulting in higher hydrogen evolution. When increasing the photocatalyst concentration to higher than the optimized amount, the activity of the photocatalysts decreased due to decreasing the exposure/penetration of light to the catalysts. At higher concentration of photocatalyst, the particles agglomerate and block the sunlight irradiation. A 5 mg dose of photocatalyst gave the optimum yield of hydrogen, which was 7.05 mmol g^−1^. When using 10 mg dose of photocatalysts, only 9.02 mmol g^−1^ hydrogen was recorded. See the results in Fig. 9(b).

### pH

Hydrogen evolution also depends on the concentration of protons (H^+^), which refers to the pH of the solution.^57,58^ These protons are reduced by accepting the electrons that are generated by photocatalysts to effect the conversion into hydrogen gas.^59^ The hydrogen-evolution rates in acidic and basic media are different, and the maximum hydrogen was produced in a weak basic medium at pH 10.^60^ Photocatalytic reactions were thus carried out at pH 10 and generated a maximum hydrogen evolution of 7.03 mmol g^−1^, as shown in Fig. 9(c). In this work, it was found that at high and low pH, the hydrogen-evolution rate decreased, generally because in highly acidic and basic reaction media, the stability of a photocatalyst is affected and it is reduced.^61^ And as a result, the activity of the photocatalysts for hydrogen production is retarded. The optimum conditions for a high rate of hydrogen evolution were recorded with an aqueous medium at pH 10.

### Temperature

Temperature does not have significant effect on the activity of photocatalysts and it cannot induce electrons thermodynamically to promote the generation of hydrogen. However, it affects the desorption of reactive products at the surfaces of the photocatalysts.^62^ Due to this higher desorption, the activity of photocatalysts for hydrogen generation can be significantly increased.^63^ At high temperature, the desorption process speeds up, so more hydrogen can be produced at reaction sites, whereas at low temperature, the desorption process slows down and products accumulate on the surface of photocatalysts. Although, a high temperatures increases the rate of transfer of electrons from the valence band to the conduction band of a semiconductor and facilitates electron–hole formation,^64^ at the same time, it can enhance the vapour pressure, which can affect the efficiency of the photoreactions.^65^ The optimum temperature for the evolution of hydrogen was found to be about 60 °C.^66^ At 60 °C, the hydrogen-evolution rate was 7.02 mmol g^−1^, as shown in Fig. 9(d). It has been reported that at high temperature, the stability and phase morphology of the catalysts may altered.^67,68^ Photocatalysts prepared without calcination produced less hydrogen compared to the catalysts that were calcined during their preparation. The photocatalysts prepared at different calcination temperatures had different sizes and structures.^52^

### Role of the sacrificial reagent

Photocatalysts used for water splitting to produce hydrogen at large scale have several problems, one being the recombination of holes and electrons produced by photocatalytic reactions.^69,70^ To avoid the recombination of electrons, sacrificial reagents can be used to consume the holes produced during photoreactions at the valence band of photocatalysts.^71^ Electrons absorb energy and excite towards the conduction band of the catalysts and are there utilized for the generation of hydrogen as a result of water reduction. Holes created in the valence band are absorbed by the sacrificial reagent and converted into oxidized products. To absorb one type of charge carrier at a high rate as compared to other types, the following reagents could be preferably utilized: Ag^+^, Fe^3+^, and Ce^4+^ for the oxidation of water by accepting electrons to enhance the production of oxygen gas.^72^ Ethanol, methanol, lactic acid, tri ethanol amine, cyanide, EDTA, Na_2_SO_3_, and Na_2_S can be used as electron donors to consume holes and produce hydrogen gas from the reduction of water. Ethanol and methanol are good sacrificial reagents. In this research work, 5% ethanol was used as a sacrificial agent, because it was comparatively cheaper than the other options. The other reason was that alcohols can be easily obtained from renewable sources. Ethanol consumes holes on the catalysts surfaces for generating oxidized products.^73^ The concentration of sacrificial reagent used for the scavenging of electrons also affects the hydrogen production. Here, 5% ethanol as a sacrificial reagent was found to be the optimized amount. As we increased the ethanol concentration up to 25%, the rate of hydrogen production lightly increased. When increasing the concentration of sacrificial reagent too high, the surfaces of the photocatalysts became saturated and impeded the activity.

## Conclusions

In general, zinc vanadate photocatalysts (Zn_3_V_2_O_8_) were prepared by a co-precipitation method followed by hydrothermal treatment to obtain a better morphology of catalysts. Various amounts of Au metals (overall 1.0% w/w) were deposited over Zn_3_V_2_O_8_ support surfaces to evaluate the hydrogen-production activities. The purity, structural morphology and elemental composition of catalysts were confirmed by XRD and EDX analysis. An orthorhombic crystalline structure with a 14.90 nm crystallite size of zinc vanadate was revealed by X-ray diffraction. The hydrogen-generation rates over various Au@Zn_3_V_2_O_8_ photocatalysts were examined. Overall, 7.05 mmol g^−1^ h^−1^ H_2_ generation was obtained for the most active catalysts of the series (*i.e.* Au_1.0_@Zn_3_V_2_O_8_), which was ten times higher than bare Zn_3_V_2_O_8_ (0.78 mmol g^−1^ h^−1^). It was found that the hydrogen-production activity of Zn_3_V_2_O_8_ could be remarkably enhanced by employing metallic Au nanoparticles. The higher photocatalytic activities were attributed to the plasmonic-induced electrons of Au generating Schottky junctions at the point of contact between the metal and semiconductor system. The recyclability tests revealed that the hydrogen evolution consistently increased due to the inherent stability of the photocatalysts. Several factors, namely, method of preparation, particle size, calcination time and morphology of the catalysts influence the efficiency of catalysts. By improving these factors, efficiency of Zn_3_V_2_O_8_ could be enhanced for photocatalytic applications.

## Conflicts of interest

The authors declare no competing financial interest.

## Supplementary Material

NA-005-D3NA00148B-s001
