# Supplementary material for: Facile transfer of surface plasmon electrons of Au-NPs to Zn3V2O8 surfaces: a case study of sunlight driven H2 generation from water splitting
Source: Nanoscale Adv. 2023 Apr 11;5(12):3233–46. doi: 10.1039/d3na00148b (PMC10263007; doi:10.1039/d3na00148b)
Supplement: NA-005-D3NA00148B-s001 [file NA-005-D3NA00148B-s001.pdf]

Electronic Supplementary Material (ESI) for Nanoscale Advances.
This journal is © The Royal Society of Chemistry 2023

Supporting information

Facile transfer of surface plasmon electrons of Au-NPs to $Zn_3V_2O_8$ surfaces: A case study of sun-light driven H_2 generation from water splitting†

Muhammad Jalil[a], Khezina Rafiq[a]*, Muhammad Zeeshan Abid[a], Abdul Rauf[a], Shuxin Wang[b], Shahid Iqbal[c] and Ejaz Hussain[a]*

[a]Institute of Chemistry, Inorganic Materials Laboratory 52S, The Islamia University of Bahawalpur–63100, Pakistan
[b]College of Materials Science and Engineering, Qingdao University of Science and Technology, Qingdao–266042, P.R. China
[c]Department of Physics, Illinois Wesleyan University, IL–61702–2900, USA

Table S1: Crystallite size calculation by applying Scherer formula

Catalyst	FWHM	2θ	D (nm)	Eg (eV)
$Zn_3V_2O_8$	0.5848	36.5270	14.95	3.0
Au@$Zn_3V_2O_8$	0.4645	36.2070	18.80	2.9

Scherer Formula: To calculate the crystallite size of $Zn_3V_2O_8$ and Au@$Zn_3V_2O_8$ photocatalysts Scherer equation is used which is given below.

$$D = \frac{k\lambda}{\beta\cos\theta}$$

Where D is crystallite size, k is Scherer constant which is equal to 0.9, λ is wavelength which is equal to the 0.15, β is full width at half maximum (FWHM) of peaks and θ is the angle of diffraction.

Table S2: XRD parameters of $Zn_3V_2O_8$

JCPDS # PDF 34-0378 $Zn_3V_2O_8$ Orthorhombic				
**Sr. No.**	**2-Theta**	**Theta**	**d(Å)**	**(h k l)**
1	15.365	7.682	5.7620	(0 2 0)
2	18.741	9.370	4.7310	(1 2 0)
3	26.450	13.225	3.3670	(2 2 0)
4	27.080	13.540	3.2900	(2 1 1)
5	29. 454	14.727	3.0300	(1 3 1)
6	31.004	15.502	2.8820	(0 4 0)
7	34.910	17.455	2.5680	(1 2 2)
8	35. 980	17.990	2.4940	(3 2 0)
9	36.464	18.232	2.4620	(3 1 1)
10	43.114	21.557	2.0964	(0 4 2)
11	43.301	21.650	2.0878	(1 5 1)
12	48.606	24.303	1.8716	(1 6 0)
13	57.703	28.851	1.5963	(1 6 2)
14	58.440	29.220	1.5779	(3 6 0)
15	60.562	30.281	1.5276	(0 0 4)
16	62.973	31.486	1.4748	(4 4 2)
17	64.606	32.303	1.4414	(0 8 0)

Table S3: Comparison of metal vanadates photocatalysts for hydrogen production reported in literature.

**Catalyst**	**Light source**	**Catalyst amount**	**Sacrificial reagent**	**H_2 production**	**Ref.**
Au@$Zn_3V_2O_8$	sunlight	5 mg	5 % Ethanol	7.50 $mmolg^{-1}h^{-1}$	Present study
$BiVO_4/Ti_3C_2$	〃	〃	-	0.15 $mmolg^{-1}h^{-1}$	[1]
$CeO_2/CeVO_4/V_2O_5$	〃	〃	-	0.18 $mmolg^{-1}h^{-1}$	[2]
Cr:$Cu_3V_2O_8$	〃	〃	5 % NaI	0.28 $mmolg^{-1}h^{-1}$	[3]

Table S4: Recyclability test for most active photocatalysts ($Au_{1.0}@Zn_3V_2O_8$).

Photocatalyst ($Au_{1.0}@Zn_3V_2O_8$)	Time (h)						H_2 generation
	1	2	3	4	5	6	
1st run	3.59	10.56	17.83	25.40	33.14	41.14	mmol g^{-1}
2nd run	3.58	10.30	17.50	24.80	32.30	40.20	〃
3rd run	3.41	10.16	17.34	24.07	31.64	39.14	〃

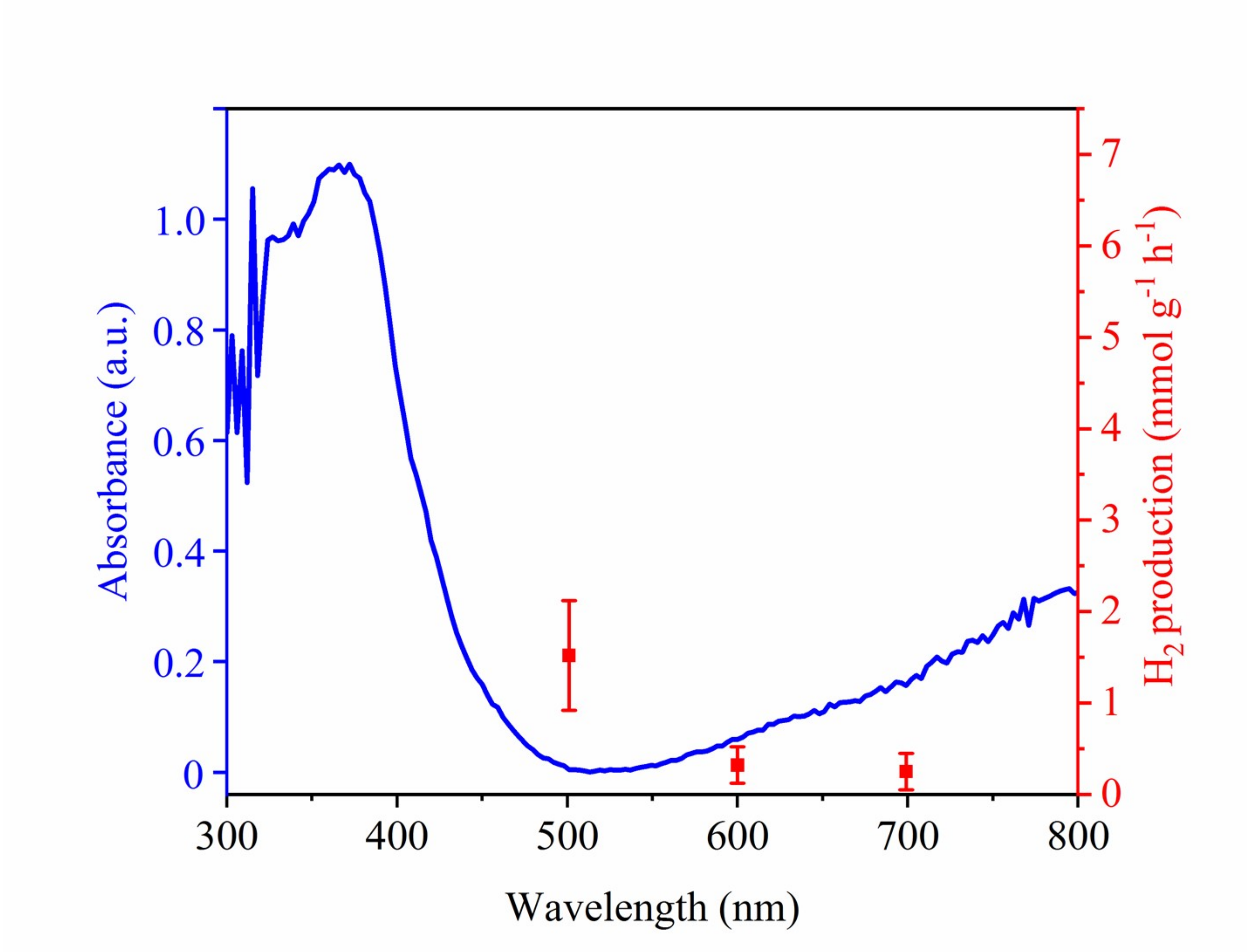

Figure S1: Visible light induced SPR effect of Au metal contents over $Zn_3V_2O_8$ for H_2 productions.

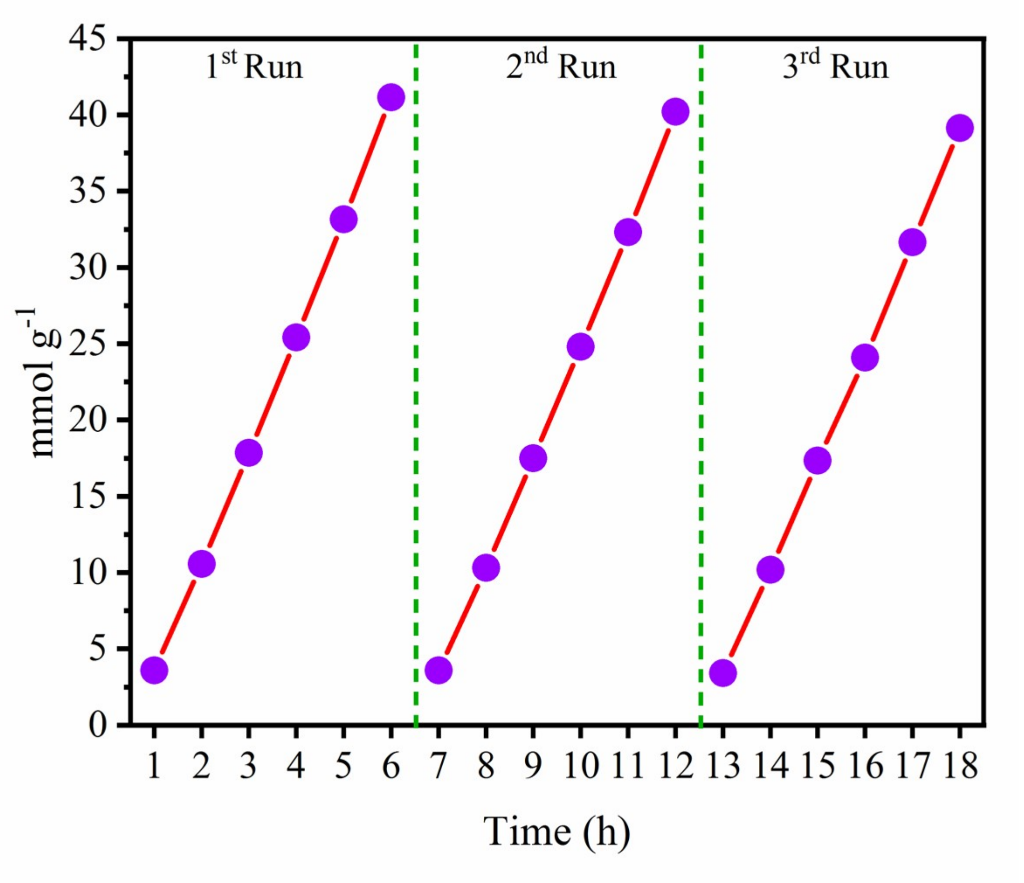

Figure S2: Recyclability test of Au@$Zn_3V_2O_8$ photocatalysts.

Reference:

1. Li, Y., et al., *2D/2D heterostructure of ultrathin $BiVO_4/Ti_3C_2$ nanosheets for photocatalytic overall Water splitting.* Applied Catalysis B: Environmental, 2021. **285**: p. 119855.
2. Cui, X., et al., *Self-generating $CeVO_4$ as conductive channel within $CeO_2/CeVO_4/V_2O_5$ to induce Z-scheme-charge-transfer driven photocatalytic degradation coupled with hydrogen production.* International Journal of Hydrogen Energy, 2019. **44**(43): p. 23921-23935.
3. Cárdenas Morcoso, D., *Chromium-doped copper vanadate films (Cr: $Cu_3V_2O_8$) as photoanodes for water splitting.* 2017.
